# Impact of assessment and treatment of neuropathic pain in patients with chronic diabetic neuropathy assisted in a diabetes reference service

**DOI:** 10.1186/1758-5996-7-S1-A34

**Published:** 2015-11-11

**Authors:** Carolina Ritzmann, Lucia Henriques Alves da Silva, Rosane Kupfer

**Affiliations:** 1 Instituto Estadual de Diabetes e Endocrinologia Luiz Capriglione, Rio de Janeiro, Brazil

## Background

Diabetic neuropathy is one of the chronic complications of hyperglycemia, which characterizes Diabetes Mellitus. As a result of an indolent course, the absence of signs and symptoms and nonspecific manifestations, it can remain undiagnosed for a long time. One of the clinical presentations is the presence of neuropathic symptoms, such as pain, cramps or paresthesia, which can compromise the quality of life.

## Objective

The objective of this study was to demonstrate that the pharmacological treatment of neuropathic pain can promote a significant symptomatic improvement assessed by validated scores and that the establishment of a support center in neuropathy constitutes an important measure in the context of multidisciplinary care of the diabetic patient.

## Materials and methods

53 patients were recruited with a mean age of 58 yrs., similar gender distribution (50.9% male and 49.1% female), mostly with type 2 Diabetes Mellitus (83%), average diagnosis time of 18 yrs. and median time for neuropathic symptoms of 2 yrs.

## Results

At the first visit, subjects had pain intensity classified as moderate by the Analog Pain Scale, intensity of neuropathic symptoms classified as severe (56.6%) and neuropathic disability rated as moderate to severe impairment (41.5%). Following the prescription of specific pharmacological treatment, 91.6% of patients reported maintenance or improvement of symptoms and only 2.8% reported worsening. It was observed significant improvement in Neuropathic Symptoms Score (p=0.0006) and Analogic Pain Scale (p <0.0001), especially in type 2 diabetic group. There was no change in Neuropathic Disability Score.

## Conclusion

This study demonstrated that the existence of a support center for assessment and treatment of painful diabetic neuropathy in a Diabetes reference service allows early diagnosis and intervention in neuropathic symptoms in an effective way.

**Figure 1:**
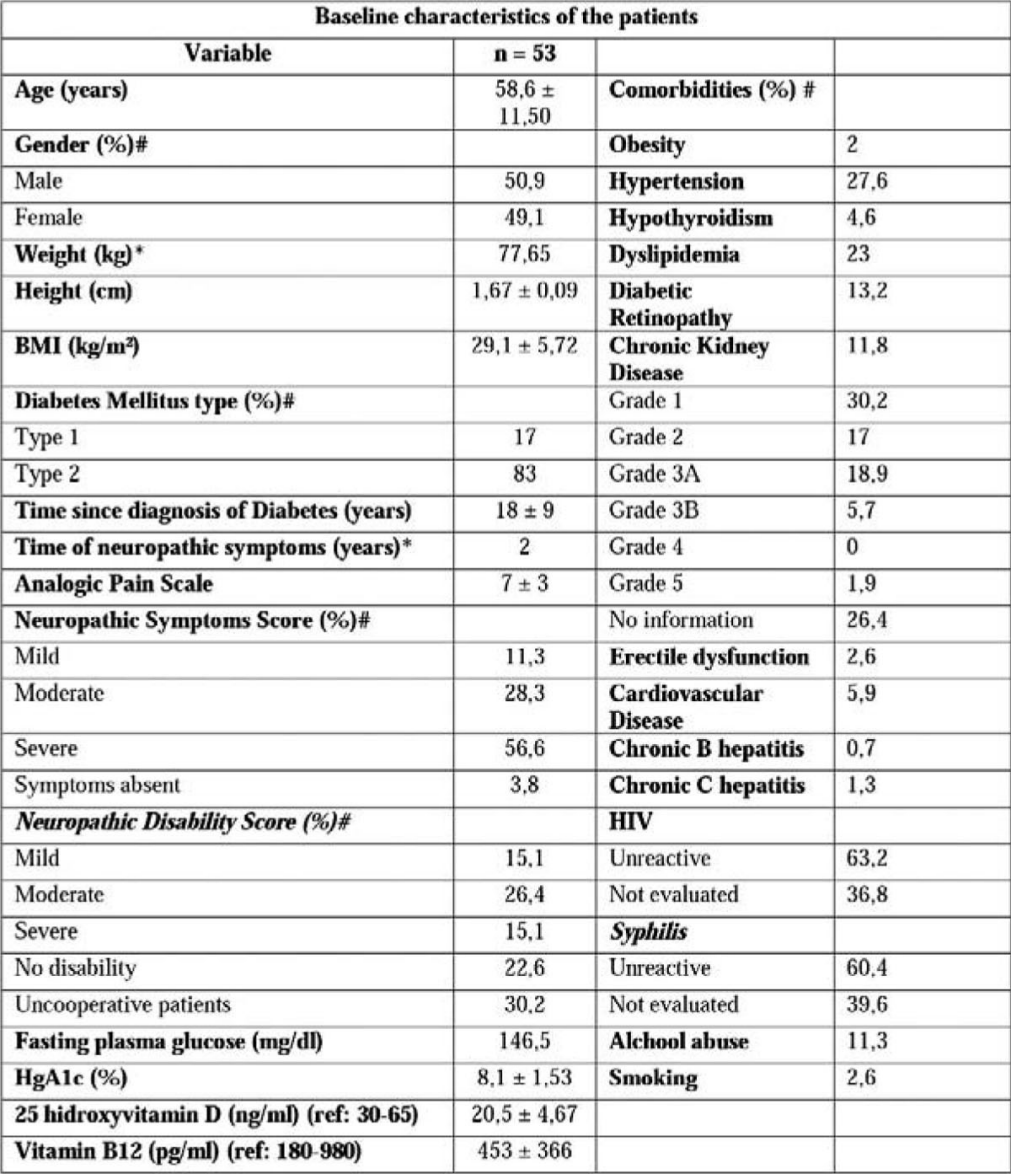
**Baseline characteristics of the patients.** # values expressed in relative frequency; Analogic Pain Scale: 0-2: mild; 3-7 moderate; 8-10: severe; Neuropathic Symptoms Score: 1-4: mild; 5-6 moderate; 7-10: severe; Neuropathic Disability Score 3-5: mild; 6-8: moderate; 9-10: severe; Uncooperative patients: patients in whose Achilles reflex couldn't be assessed; No information: information not find at the records.

**Figure 2:**
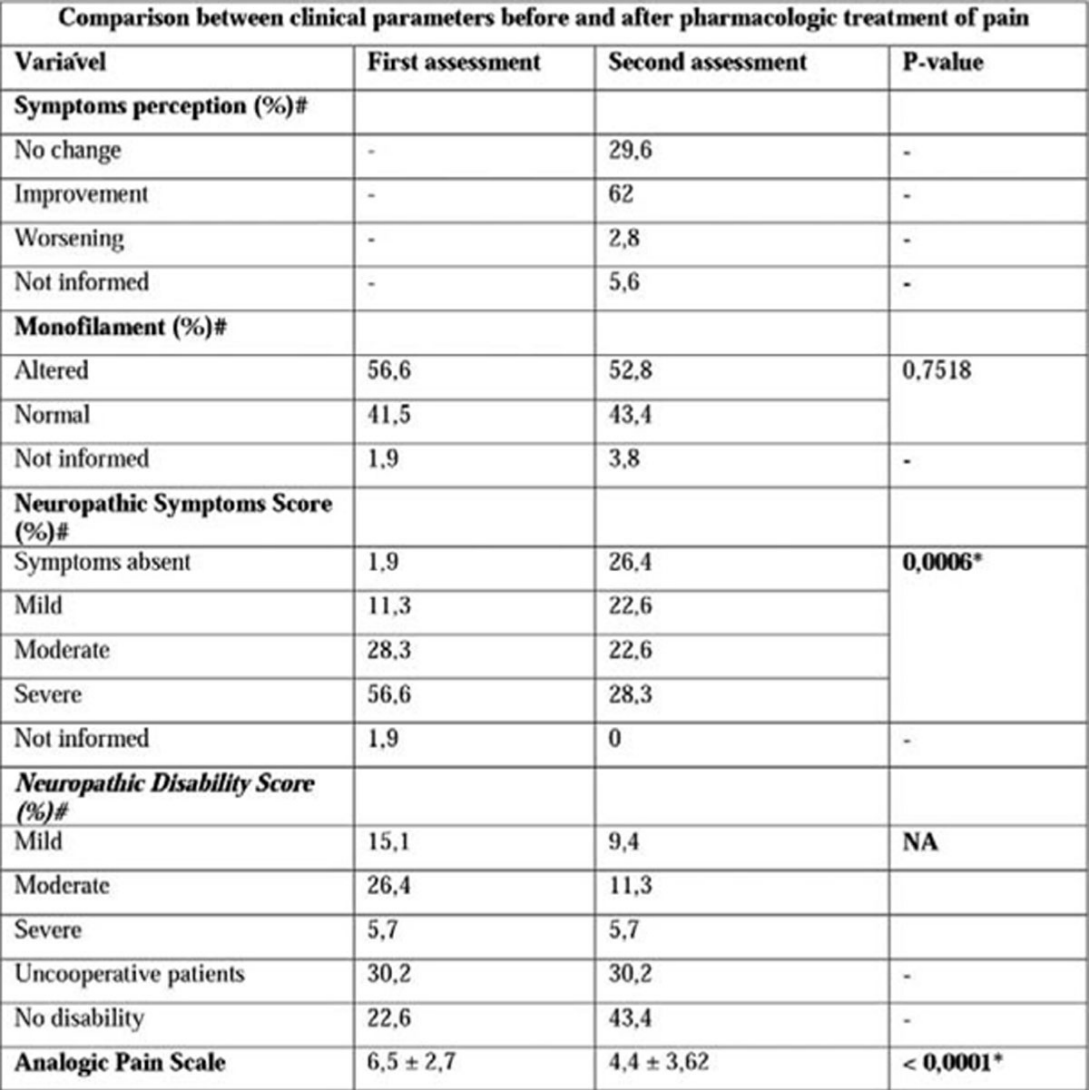
**Comparison between clinical parameters before and after pharmacologic treatment of pain.** *statistically significant P-value (< 0.05); # values expressed in relative frequency; Analogic Pain Scale: 0-2: mild; 3-7 moderate; 8-10: severe; Neuropathic Symptoms Score: 1-4: mild; 5-6 moderate; 7-10: severe; Neuropathic Disability Score 3-5: mild; 6-8: moderate; 9-10: severe; Uncooperative patients: patients in whose Achilles reflex couldn't be assessed; No information: information not find at the records

